# Effect of *Fomes fomentarius* Cultivation Conditions on Its Adsorption Performance for Anionic and Cationic Dyes

**DOI:** 10.1021/acsomega.1c05748

**Published:** 2022-01-24

**Authors:** Laura M. Henning, Ulla Simon, Amanmyrat Abdullayev, Bertram Schmidt, Carsten Pohl, Tamara Nunez Guitar, Cekdar Vakifahmetoglu, Vera Meyer, Maged F. Bekheet, Aleksander Gurlo

**Affiliations:** † Chair of Advanced Ceramic Materials, Institute of Material Science and Technology, Faculty III Process Sciences, Technische Universität Berlin, Straße des 17. Juni 135, 10623 Berlin, Germany; ‡ Chair of Applied and Molecular Microbiology, Institute of Biotechnology, Faculty III Process Sciences, Technische Universität Berlin, Straße des 17. Juni 135, 10623 Berlin, Germany; § Department of Materials Science and Engineering, Izmir Institute of Technology, Urla, 35430 Izmir, Turkey

## Abstract

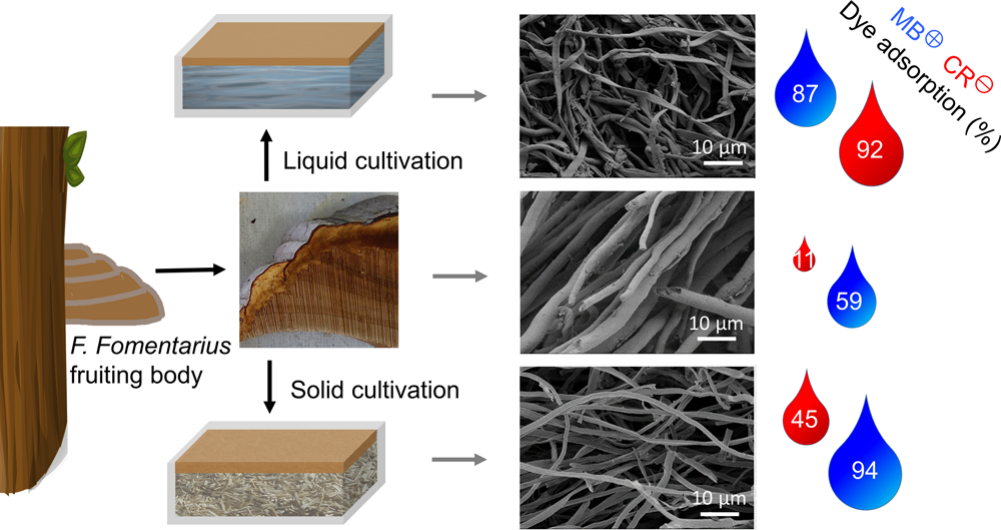

Lab-cultivated mycelia of *Fomes fomentarius* (FF), grown on a solid lignocellulose medium (FF-SM) and a liquid glucose medium (FF-LM), and naturally grown fruiting bodies (FF-FB) were studied as biosorbents for the removal of organic dyes methylene blue and Congo red (CR). Both the chemical and microstructural differences were revealed using X-ray photoelectron spectroscopy, Fourier-transform infrared spectroscopy, zeta potential analysis, and scanning electron microscopy, illuminating the superiority of FF-LM and FF-SM over FF-FB in dye adsorption. The adsorption process of CR on FF-LM and FF-SM is best described by the Redlich–Peterson model with β constants close to 1, that is, approaching the monolayer Langmuir model, which reach maximum adsorption capacities of 48.8 and 13.4 mg g^–1^, respectively, in neutral solutions. Adsorption kinetics follow the pseudo-second-order model where chemisorption is the rate-controlling step. While the desorption efficiencies were low, adsorption performances were preserved and even enhanced under simulated dye effluent conditions. The results suggest that *F. fomentarius* can be considered an attractive biosorbent in industrial wastewater treatment and that its cultivation conditions can be specifically tailored to tune its cell wall composition and adsorption performance.

## Introduction

Industrial dye effluents from various industries such as textile, leather, plastic, food, pharmaceutical, cosmetics, or paper printing industries cause severe threats to the ecosystem when discharged into the environment.^1−4^ Approximately 15% of the original dye is lost during the processing in the textile industry.^1,2^ Dyes are not easily biodegradable. Often, they are highly toxic or even carcinogenic to aquatic life or human beings. Furthermore, even small concentrations of dyes can lead to water turbidity and cause a decrease in the permeability of the sunlight, which may reduce the photosynthesis and hinder the life of aquatic organisms.^5^

Industrial wastewater can be purified by several methods such as membrane separation, chemical precipitation, oxidation/reduction, and adsorption. However, large amounts are still disposed to the environment without purification. An accurate estimation is difficult because of data inaccessibility but is still reported to reach up to 50% worldwide.^6^ Because of its simplicity, cost-effectiveness, high removal efficiencies, and operational ease, adsorption is an attractive wastewater treatment approach.^7−9^ New classes of adsorbent materials derived from natural materials, industrial solid wastes, renewable materials, agricultural byproducts, and organisms such as bacteria, fungi, algae, and seaweeds are increasingly in focus from an environmental perspective as these potentially biodegradable materials reduce the environmental pollution and waste.^10,11^

Different genera of filamentous fungi belonging to Ascomycota and Basidiomycota can be adopted for dye biodegradation and biosorption from wastewater.^5,11−14^ Various mechanisms play a role in dye removal: (i) biosorption of the dye onto the cell wall surface, (ii) biodegradation of dye molecules by ligninolytic enzymes such as laccases and peroxidases, and (iii) bioaccumulation of the dye in living cells by transport membrane systems.^14,15^ Living filamentous fungi are particularly suitable for biodegradation and bioaccumulation processes, while biosorption can occur by both living and dead mycelia. Fungal cell walls mainly consist of α-glucans, β-glucans, chitin, galactomannans, and glycoproteins and thus provide high amounts of surface functional groups such as amine, carboxyl, hydroxyl, phosphate, sulfhydryl, amino, amide, and epoxy groups, enabling physicochemical interactions with organic dyes or other toxic molecules and thus enabling biosorption phenomena.^14,16^ Notably, the use of dried mycelium offers simpler and less costly biosorption processes as the physicochemical process conditions can be freely selected over a wide range. Furthermore, dried mycelium can be kept for a long time under appropriate storage conditions with only minor changes in properties. Regeneration through desorption and thus further use of fungal biosorbents, as well as nutrient recovery by composting, offer additional room for innovation and application.

Besides basidiomycetes of orders Polyporales and Hymenochaetales, including *Phanerochaete chrysosporium*, *Inonotus dryadeus*, *Trametes versicolor*, *Daedalea dickinsii*, *Daedalea africana*, and *Phellinus adaman*,^3,15,17−26^ ascomycetes such as *Aspergillus carbonarius*,^27,28^*Penicillium glabrum*,^27,28^ and *Aspergillus niger*(17,29) were studied for the removal of various dyes. Fruiting bodies of polypore *Fomes fomentarius* were investigated as potential biosorbents for dye removal.^20,21^ Furthermore, purified and cross-linked enzyme aggregates obtained from submerged cultures of *F. fomentarius* were found to be promising candidates for dye degradation.^30^

The cell wall composition differs among fungi and even changes dynamically in a single fungal species during its life cycle. It depends on the nutrient sources available, the cultivation method, the metabolic activity and age of the mycelium, its branching rate, and its cell wall stress response when confronted with (sub)lethal concentrations of toxic compounds that inhibit cell wall biosynthesis, such as Congo red (CR) or other antifungals.^31^ The cell wall composition can also strongly vary between strains of the same fungal species.^32−36^ None of these genetic and physiologic factors have been studied so far in the context of understanding and improving biosorption characteristics of living or dried fungal mycelia.

Accordingly, in the current work, the biosorption properties of *F. fomentarius* mycelium cultivated under laboratory conditions as an emersed culture, that is, on a solid medium or a liquid medium, are compared to those of naturally grown fruiting bodies. Exemplarily, the adsorption of the cationic dye methylene blue (MB) and the anionic dye CR from aqueous solutions is investigated.

To understand the sorption capacities of the tinder polypore *F. fomentarius* and to provide a basis for their future optimization by genetic and cultivation means, a native strain was isolated from a dead tree trunk from the Brandenburg forest (Germany), and an axenic culture was obtained from it. This fungal species is of interest as it is well-known in traditional medicine as a vital fungus and can be used for the production of wound-healing textiles as well as composite materials for the construction industry.^37,38^*F. fomentarius* grows well under laboratory conditions on different byproducts from agriculture and forestry including hemp, raps straw, and sawdust and thus could potentially become a future cell factory for the sustainable and customizable manufacturing of fungal-based materials exploited by different industries.

## Results and Discussion

**Figure 1:**
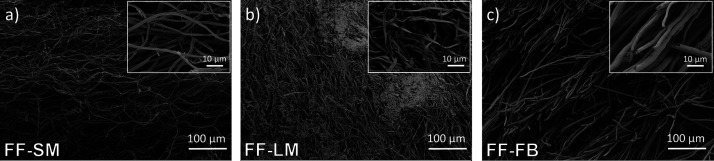
SEM images from (a) FF-SM, (b) FF-LM, and (c) FF-FB. Scale bars shown are 100 and 10 μm for the image and inset, respectively.

Visual estimation of dried *F. fomentarius* discs obtained from three different cultivations reveals differences in color and bulk densities, as can be seen in Figure S1. While *F. fomentarius* grown on a solid lignocellulose medium, referred to as FF-SM, shows the lowest bulk density and a faint beige-to-yellow color, an increase in bulk density and color intensity can be observed for *F. fomentarius* grown on a liquid glucose medium, referred to as FF-LM. Fruiting body of *F. fomentarius* collected from the nature, referred to as FF-FB, shows the highest bulk density and an intense brownish color. In accordance, scanning electron microscopy (SEM) images in Figure 1 reveal that the mycelium network of both laboratory-cultivated *F. fomentarius* samples FF-SM and FF-LM consist of loose and randomly packed hyphae with diameters of ca. 2–3 μm, whereas the mycelium network of trama FF-FB is composed of more aligned and densely packed hyphae with diameters of ca. 6–7 μm. Furthermore, FF-FB shows cylindrical-shaped fibers, while FF-SM and FF-LM exhibit rather smashed or planar-like fibers, which may be attributed to the drying process.

**Figure 2:**
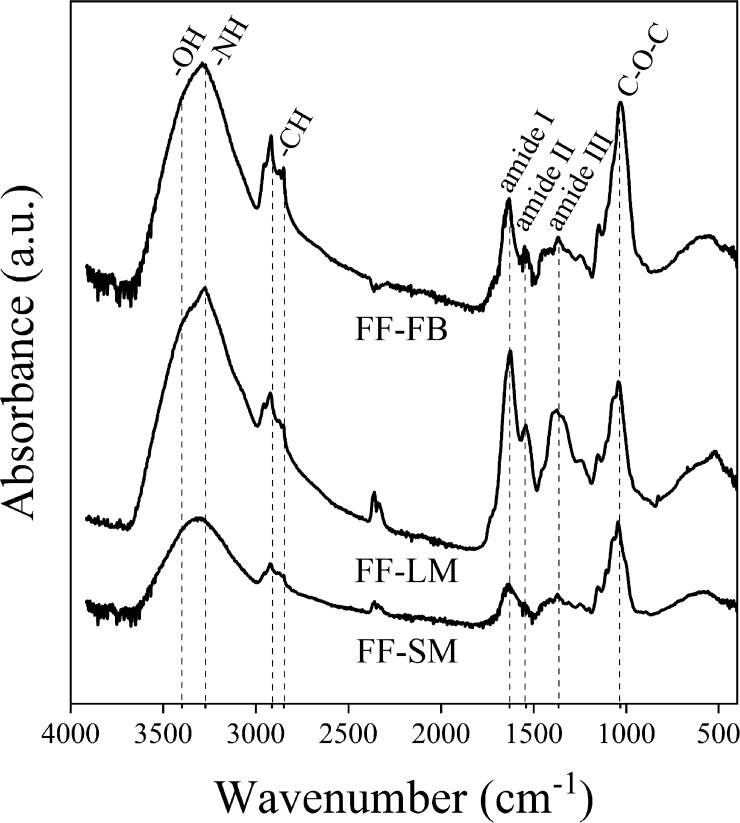
FTIR–ATR spectra of FF-SM, FF-LM, and FF-FB, showing a higher relative absorbance of amide bands in FF-LM which might be indicative of higher chitin levels.

Fourier transform infrared spectroscopy (FTIR)–attenuated total reflection (ATR) spectra of the three samples are presented in Figure 2. Independent of the cultivation protocol, all spectra show the characteristic absorption bands of the carbohydrate backbone present in both the glucan and chitin polymer structure, such as the stretching bands of −OH at 340 cm^–1^ and −NH at 3273 cm^–1^ in addition to the −CH bands at 2913 and 2848 cm^–1^ and the −C–O–C– band at 1032 cm^–1^.^39^ The presence of the amide I band associated with −C=O stretching at 1630 cm^–1^ and amide II and III bands, resulting from the −NH deformation at 1540 and 1320 cm^–1^, respectively, indicates the presence of chitin.^40,41^ Although chitin is a primary component of the cell wall in all three samples, other amino-containing components such as peptides and proteins can also be found in fungi.^42,43^ Notably, FF-LM exhibits higher relative absorbance of the amide bands in relation to the carbohydrate bands, suggesting higher chitin levels in its cell walls. The differences in the composition can be attributed to the less complex metabolism of glucose in contrast to that of lignocellulose, whose metabolic pathway requires the synergy of several enzymes, affecting the biosynthetic growth.

### Adsorption of MB and CR on *F. fomentarius*

**Figure 3:**
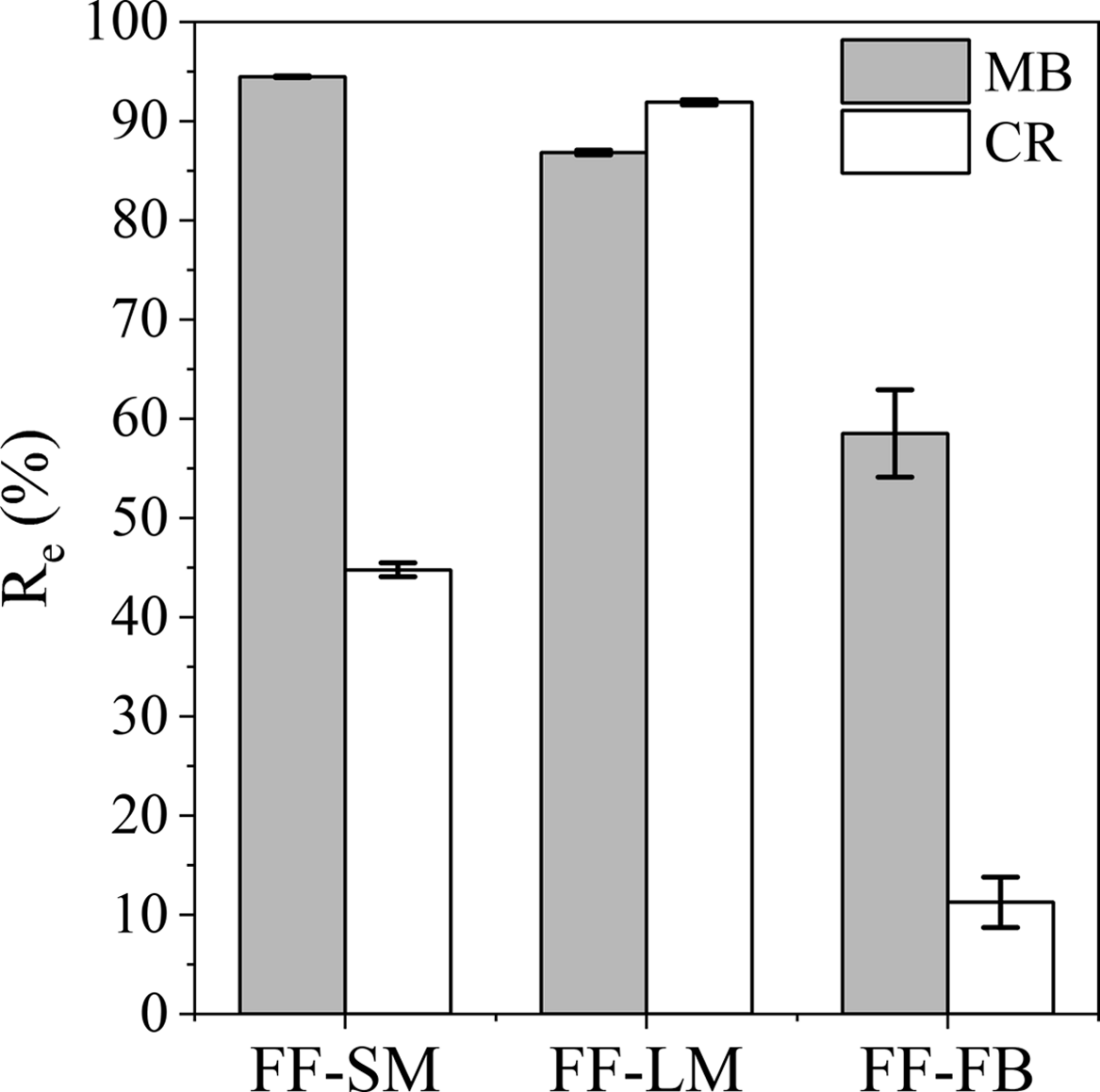
Removal efficiency *R*_e_ of MB and CR for FF-SM, FF-LM, and FF-FB. Adsorption conditions were a dosage of 5 g L^–1^, 100 mg L^–1^ MB and CR, pH values of 5.7 (MB) and 7.6 (CR), and 120 min.

The equilibrium removal efficiency (%) for all three samples FF-SM, FF-LM, and FF-FB, with an initial concentration of MB and CR of 100 mg L^–1^ and a dosage of 5 g L^–1^, at pH values of 5.7 (MB) and 7.6 (CR) at 25 °C and after a contact time of 120 min is shown in Figure 3. Photographs of the supernatants can be found in Figure S2. All samples showed good adsorption capabilities for both cationic MB and anionic CR dyes, indicating that all three mycelial samples contain both negatively and positively charged functional groups on their surfaces. However, FF-SM and FF-FB display higher removal efficiencies for MB with values of 94 and 59%, respectively, than that for CR with values of 45 and 11%, respectively, suggesting that the negatively charged functional groups are more predominant on their cell wall surfaces. In contrast, FF-LM showed a high removal efficiency of 92% for CR, which was even slightly higher than that for MB with a value of 87%, indicating that cultivation on a glucose medium results in a higher amount of positively charged groups on the cell wall surface of *F. fomentarius* at a pH of 7.6. These findings were supported by zeta potential analyses, as shown in Figure 5b, which revealed that the surface charges of FF-SM and FF-FB are more negative than that of FF-LM at a pH of 5.7, while the surface charge of FF-LM becomes highly positive at a pH of 7.6. The difference in the surface charge between the samples might be explained by the higher chitin content in FF-LM, as revealed by FTIR–ATR characterization, see Figure 2. However, differences in branching frequencies, hyphal diameter, and surface area are further influential factors that affect the adsorption capacity of MB and CR. As FF-FB showed both the lowest performance and the highest standard deviation for the adsorption of MB and CR, it was not considered for further experiments. While both FF-SM and FF-LM showed high uptakes for MB, their removal efficiency differed for CR. Thus, all further experiments were conducted with CR.

### Effect of the Adsorbent Dosage

**Figure 4:**
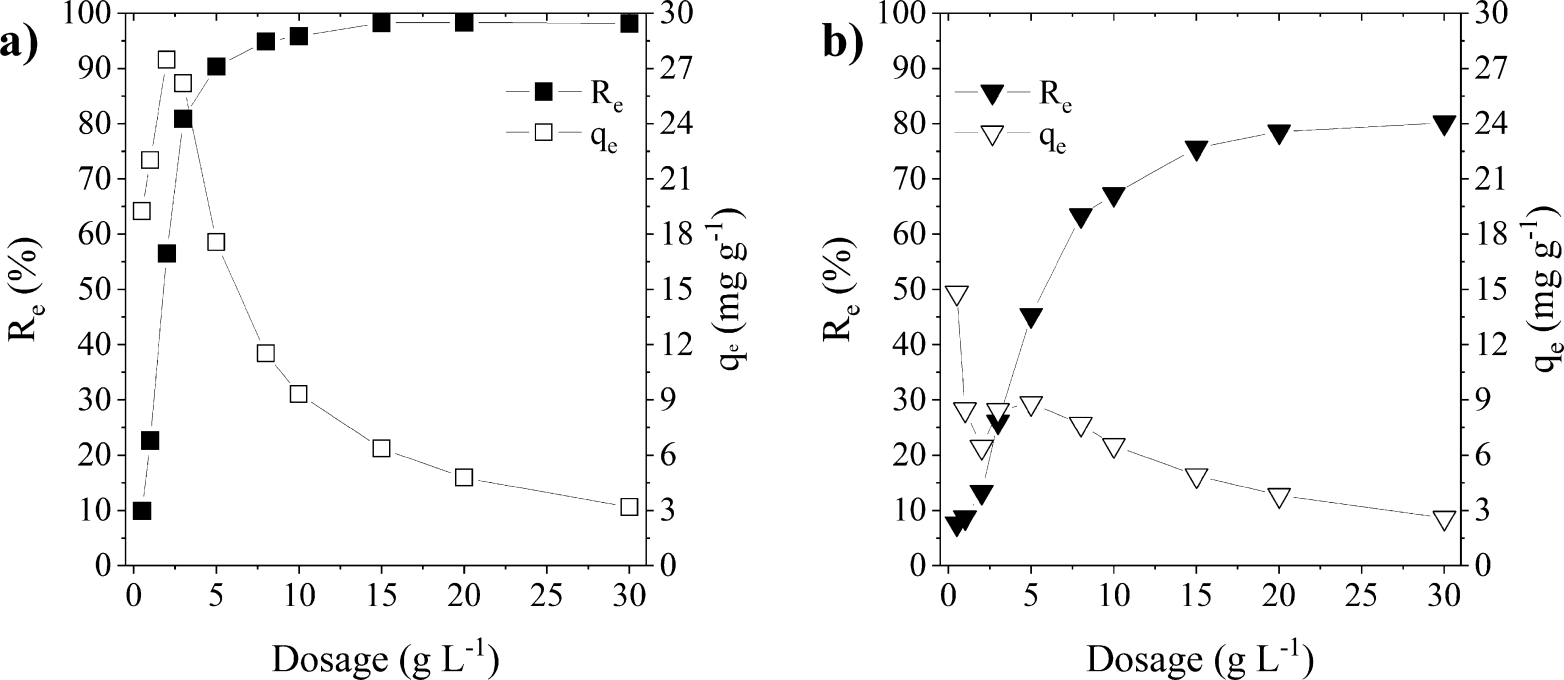
Effect of the adsorbent dosage on the removal efficiency *R*_e_ and adsorption capacity *q*_e_ of CR on (a) FF-LM and (b) FF-SM. Adsorption conditions were pH 7.6, a *C*_i_ of 100 mg L^–1^, and 120 min.

The effect of the adsorbent dosage on the adsorption process of CR with an initial concentration of 100 mg L^–1^ on FF-LM and FF-SM at a pH of 7.6 and an equilibrium time of 120 min is depicted in Figure 4. Although both laboratory-cultivated mycelial samples show an increase in the removal efficiencies with dosages in the range between 0.5 and 2 g L^–1^, their equilibrium adsorption capacities *q*_e_ behave differently. While *q*_e_ increases for FF-LM, it decreases for FF-SM in this dosage range. In greater detail, the removal efficiency of FF-LM increases from 9.9 to 56.5% in the given dosage range, which is higher than the increased amount in the dosage, resulting in an increase in *q*_e_ from 19.2 to 27.5 mg g^–1^. In contrast, a slight increase from 7.6 to 13.3% is determined for FF-SM, which is lower than the increase in the dosage amount, yielding a decrease in *q*_e_ from 14.8 to 6.4 mg g^–1^. This difference could be due to the higher content of amides on the surface of FF-LM, see Figure 2, and its different morphology and fiber diameter, see Figure 1, probably leading to a higher number of active adsorption sites on the surface for FF-LM than that on FF-SM. The aforementioned reasons can also explain the higher removal efficiency of 98.2% for FF-LM than that for FF-SM with a value of 75.6% at a dosage of 15 g L^–1^, indicating that not all adsorption sites of the FF-SM sample were saturated at this high adsorbent dosage. For dosages above 4 g L^–1^, both mycelial samples show a slight increase in the removal efficiency and a high decrease in the equilibrium adsorption capacity until constant removal efficiencies of 98.2 and 78.5% are reached at dosages of 15 and 20 g L^–1^ for FF-LM and FF-SM, respectively. The decrease in adsorption capacity until reaching constant removal efficiencies at higher dosages is due to the limited concentration of dye molecules needed to saturate all the active adsorption sites at these high adsorbent dosages.^44^ All following experiments were conducted using the adsorbent dosage of 5 g L^–1^ because both mycelial samples show considerable removal efficiencies and adsorption capacities at this adsorbent dosage.

Notably, the CR dye solution did not turn clear after the dye uptake, rather the color turned from red to orange to yellow for both mycelial samples, see Figure S3. This might be due to the release of a colored metabolite from *F. fomentarius* when dissolved in water. Photographs and UV–vis spectra of *F. fomentarius* supernatants obtained after mixing with aqueous solutions of varying pH, that is, without any dye present, are displayed in Figures S4–S6, showing yellow coloring, the strongest for FF-LM, and increased absorbance from 500 to 300 nm. However, the nature of this compound or compounds could not be revealed by high-performance liquid chromatography mass spectrometry (HPLC-MS) analysis, although characteristic *m*/*z* rations were identified for *F. fomentarius*, see Figure S7.

### Effect of the Solution pH

**Figure 5:**
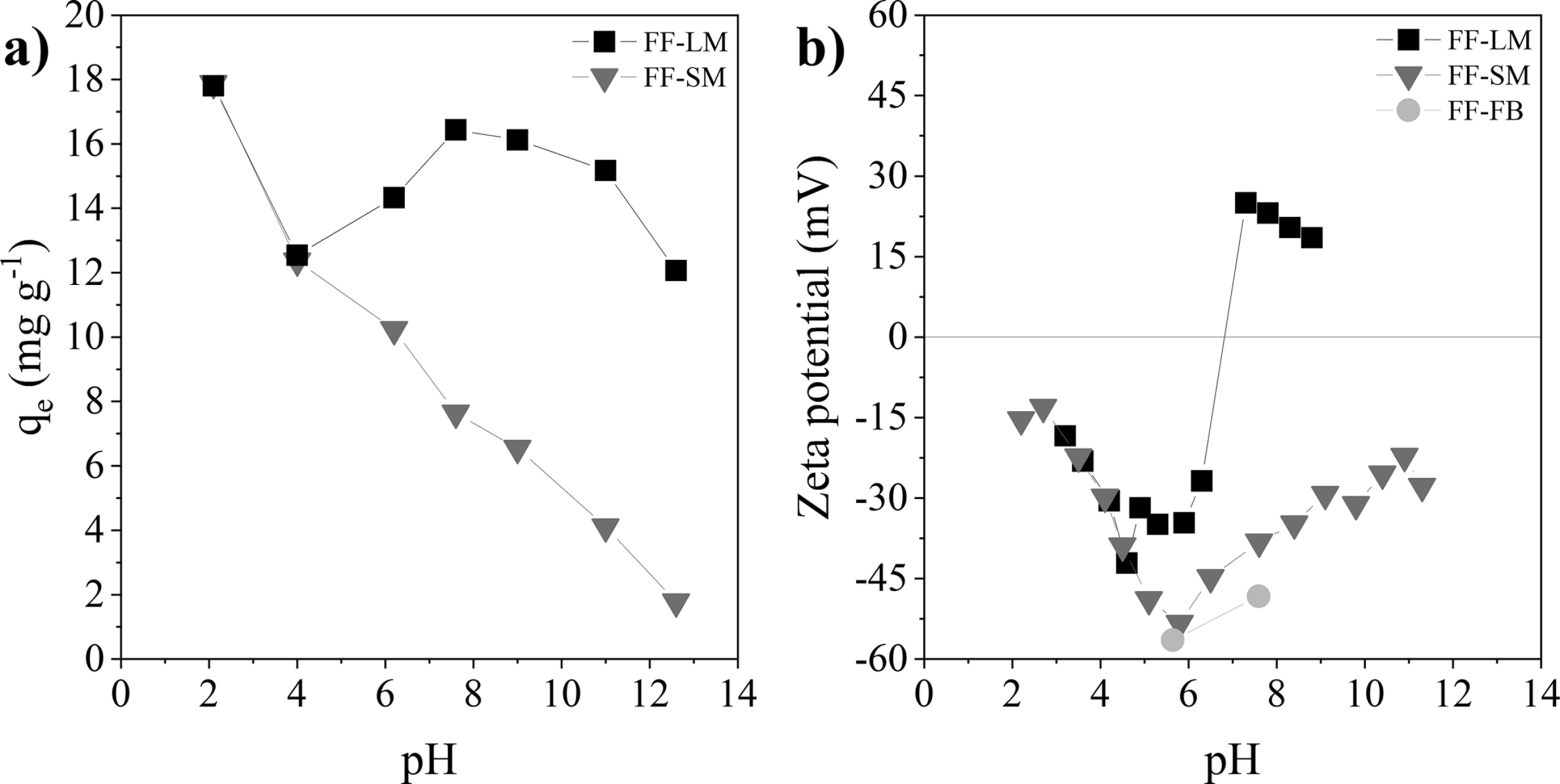
(a) Effect of the dye solution pH on the equilibrium adsorption capacity *q*_e_ of CR on FF-LM and FF-SM. Adsorption conditions were a dosage of 5 g L^–1^, a *C*_i_ of 100 mg L^–1^, and 120 min. (b) Zeta potential of FF-LM, FF-SM, and FF-FB and its pH dependence.

The adsorption performance and the dependence of the equilibrium adsorption capacity *q*_e_ on the solution pH are depicted in Figure 5. Photographs of the corresponding supernatants can be found in Figure S8. For FF-SM, *q*_e_ decreases with the increasing pH value, that is, for pH 2.1, *q*_e_ is 17.9 mg g^–1^, while it drops to 1.8 mg g^–1^ for pH 12.6. FF-LM also shows the highest *q*_e_ at low pH values, that is, 17.8 mg g^–1^ at a pH of 2.1 and the lowest *q*_e_ of 12.1 mg g^–1^ at a pH of 12.6. However, no steady decrease can be observed with increasing pH for FF-LM. Instead, at around pH 4, there is a local minimum in the adsorption capacity. Such a minimum can also be observed in the zeta potential results, see Figure 5b, indicating a more negative surface charge and thus weaker electrostatic attraction between the adsorbent and the dye. This increase in the negative surface charge with increasing pH can be explained by the deprotonation of the functional groups.^21^ However, above pH 6, the deprotonation process is hindered, and the surface of FF-LM becomes positively charged, indicating significant changes in the surface chemistry of FF-LM. These findings confirm that the growth medium significantly influences the surface chemistry. However, further characterization is needed to investigate the influence of the growth medium components on the surface charge variation at different pH values. It is worth knowing that for FF-LM, zeta potentials below pH 3 and above pH 9 could not be measured reliably and thus are not shown. For FF-SM, the zeta potential is negative over the whole pH range provided and decreases with increasing pH until a neutral pH. However, in the basic pH range, the adsorption capacity decreases further, while the zeta potential increases again, indicating that the electrostatic attraction is not the only mechanism for CR adsorption and other binding forces could potentially become more dominant, such as hydrogen bonds and van der Waals forces.^45,46^

### Adsorption Isotherms

**Figure 6:**
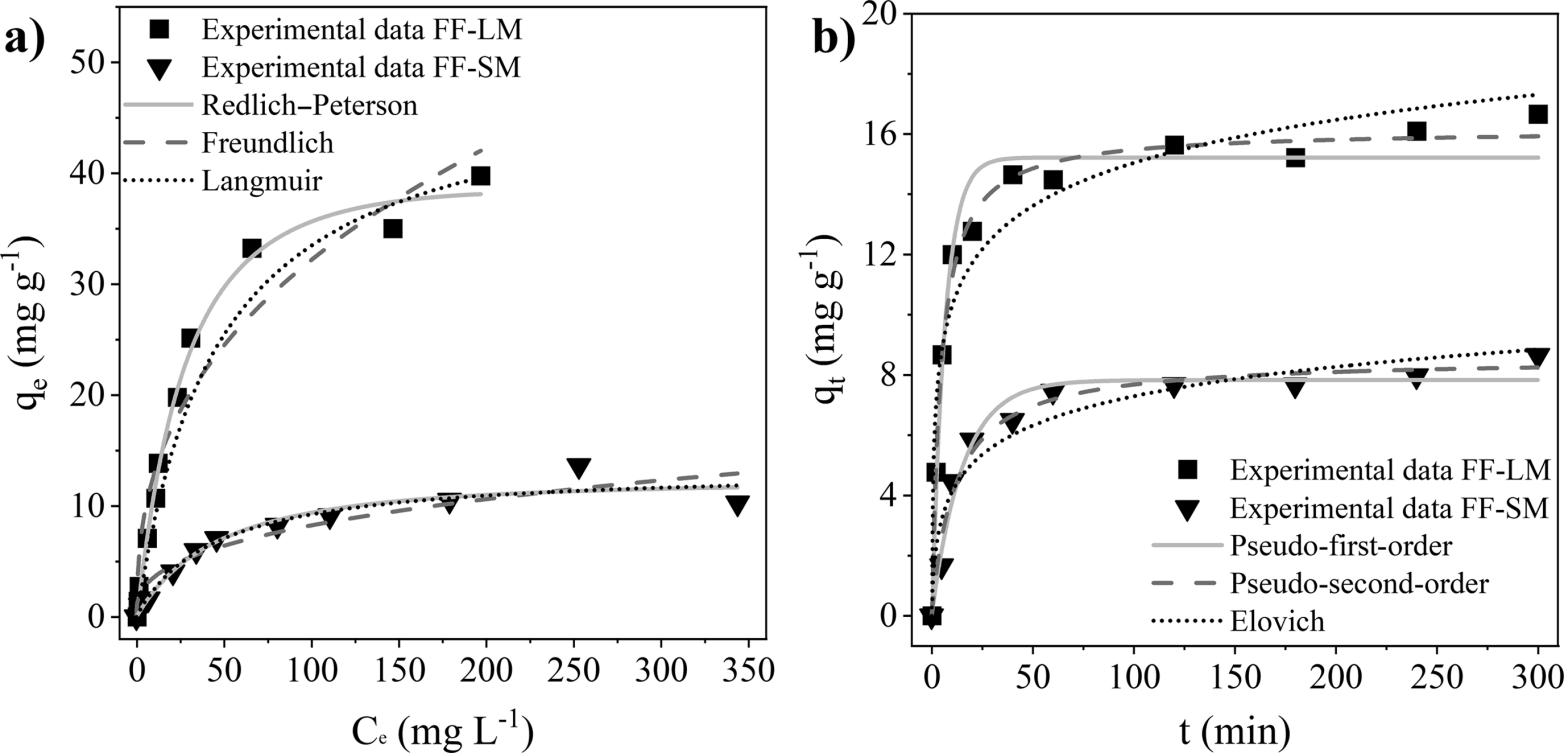
(a) Nonlinear Langmuir, Freundlich, and Redlich–Peterson isotherm models for the adsorption of CR on FF-LM and FF-SM. Adsorption conditions were pH 7.6, a dosage of 5 g L^–1^, and 120 min. (b) Nonlinear pseudo-first-order, pseudo-second-order, and Elovich kinetic fittings for the adsorption of CR on FF-LM and FF-SM. Adsorption conditions were pH 7.6, a dosage of 5 g L^–1^, and a *C*_i_ of 100 mg L^–1^.

As shown in Figure 6a and Table 1, the experimental adsorption data collected using different initial dye concentrations could be fitted the best, that is, with the highest regression coefficients, with the Redlich–Peterson model. This adsorption model combines the Langmuir model for monolayer adsorption on homogeneous sites with the Freundlich model for multilayer adsorption on heterogeneous surfaces. However, the values determined for the Redlich–Peterson constant β are close to 1, suggesting that the isotherms are approaching the Langmuir model. These results are in accordance with the higher regression coefficients obtained by fitting the adsorption data with the Langmuir model than that with the Freundlich model, see Table 1. This suggests that the adsorption of CR on the surface of laboratory-cultivated *F. fomentarius* can be described by a monolayer process rather than a multilayer process, probably due to the large size of the dye molecules, which may repel each other when getting too close. The obtained maximum adsorption capacity *q*_m_ of CR on FF-LM was found to be 48.8 mg g^–1^, which is much higher than that determined for FF-SM with a value of 13.4 mg g^–1^. However, these values are lower than those reported for other fungal species, see Table 2. On one hand, this can be explained by the lower temperature and higher dosage of 5 g L^–1^ used in this work. As shown in eq 2 and Figure 4, the equilibrium adsorption capacity decreases with the increasing dosage. On the other hand, the wide range in adsorption capacities of the fungal adsorbents listed in Table 2 can be associated with differences in the cell wall composition due to varying cultivation conditions. Likewise, the higher adsorption capacity of FF-LM over that of FF-SM reveals that the adsorption properties can be enhanced by the cultivation conditions.

**Table 1:** Parameters of the Isotherm Studies Conducted at pH 7.6, a Dosage of 5 g L^–1^, 120 min, and a *C*_i_ of 5–400 mg L^–1^ According to the Langmuir, Freundlich, and Redlich–Peterson Isotherms, as Depicted in Figure 6a, for FF-LM and FF-SM

model	parameter	FF-LM	FF-SM
Langmuir	*q*_m_ (mg g^–1^)	48.8	13.4
	*K*_L_ (L mg^–1^)	0.0219	0.0224
	*R*^2^	0.962	0.958
Freundlich	n (mg g^–1^)	2.43	2.81
	*K*_F_ (mg g^–1^ (L mg^–1^)1/n)	5.11	1.58
	*R*^2^	0.947	0.917
Redlich–Peterson	*K*_RP_ (L g^–1^)	1.30	0.27
	*a*_R_ (L mg^–1^)	0.012	0.014
	β	1.15	1.07
	*R*^2^	0.997	0.967

**Table 2:** Comparison of the Maximum Monolayer Adsorption Capacity *q*_m_ of CR on Different Dried Fungal-Based Biosorbents

species	*q*_m_ (mg g^–1^)	temperature (°C)	adsorbent dosage (g L^–1^)	pH	reference
*A. carbonarius*	99.0	30	0.3	4.5	(28)
*Aspergillus nidulans*	357.1		0.5	6.8	(47)
*P. glabrum*	101.0	30	0.3	4.5	(28)
Penicillium YW 01	357.1	20	1.0	3.0	(48)
	384.6	30			
	416.67	40			
*Agaricus bisporus*	76.4		1.0	5	(49)
*Funalia trogii*	90.4		1.0	5.0	(50)
*T. versicolor*	318.1	30	1.7	2.0	(51)
	415.7	60			
*T. versicolor*	51.8	30	30.0	7	(52)
FF-LM, *F. fomentarius* cultivated on a liquid glucose medium	48.8	25	5	7.6	this work
FF-SM, *F. fomentarius* cultivated on a solid lignocellulose medium	13.4	25	5	7.6	this work

### Adsorption Kinetics

The influence of contact time on the adsorption capacity of *F. fomentarius* mycelia at pH 7.6 with a dosage of 5 g L^–1^ and an initial CR concentration of 100 mg L^–1^ is shown in Figure 6b. The adsorption capacity increases significantly with increasing contact time before reaching equilibrium at about 1 h. The equilibrium adsorption capacity of CR on FF-LM was found to be 2 times higher than that on FF-SM. The kinetic constants and parameters as well as the nonlinear regression coefficients of fitting with the kinetic models are shown in Table 3. The experimental adsorption data of both mycelial samples were fitted better with the pseudo-second-order model when compared with that of the pseudo-first-order and Elovich kinetic model. This suggests that chemisorption is the primary rate-controlling step in the adsorption process, in which a molecule of the CR dye is adsorbed onto two sites of the cell wall surface of *F. fomentarius* at a constant concentration of the dye. However, as the pseudo-first-order model also yields high regression coefficients for both mycelial samples FF-LM and FF-SM, see Table 3, it can be concluded that physical adsorption may additionally be involved in the adsorption process.

**Table 3:** Parameters of the Kinetic Studies Conducted at pH 7.6, a Dosage of 5 g L^–1^, a *C*_i_ of 100 mg L^–1^, and 2–300 min According to the Pseudo-First-Order, Pseudo-Second-Order, and Elovich Models Depicted in Figure 6b for FF-LM and FF-SM

model	parameter	FF-LM	FF-SM
pseudo-first-order	*q*_e_ (mg g^–1^)	15.2	7.8
	*K*_1_ (min^–1^)	0.1579	0.0652
	*R*^2^	0.973	0.972
pseudo-second-order	*q*_e_ (mg g^–1^)	16.2	8.6
	*K*_2_ (g mg^–1^ min^–1^)	0.0142	0.0101
	*R*^2^	0.992	0.977
Elovich	α	30.4	2.4
	β	0.49	0.71
	*R*^2^	0.951	0.938

### Simulated Dye Effluent Adsorption

**Figure 7:**
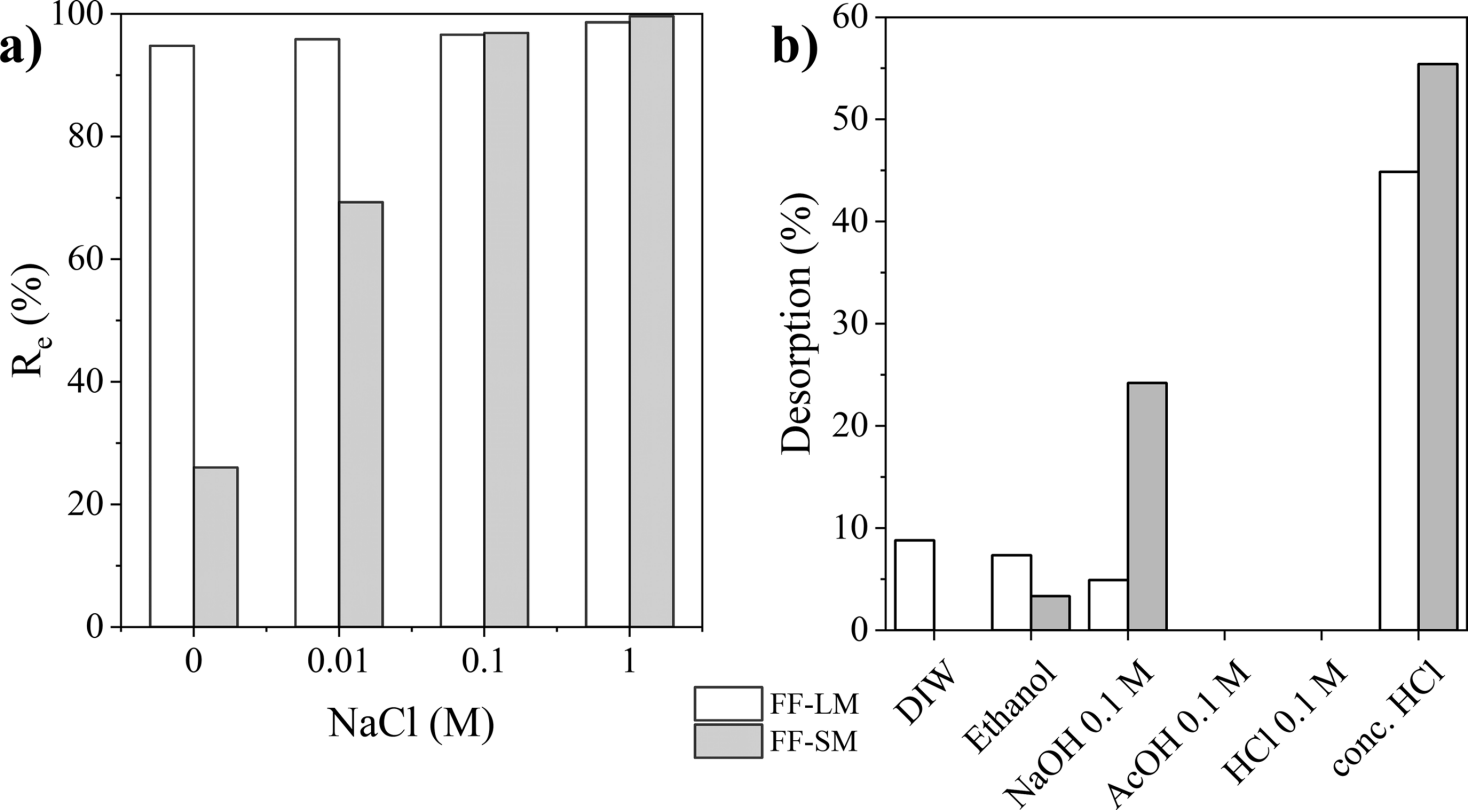
(a) Effect of simulated dye effluent conditions on the removal efficiency *R*_e_ of CR on FF-LM and FF-SM. Adsorption conditions were 80 °C, pH 7.6, a dosage of 5 g L^–1^, *C*_i_ = 100 mg L^–1^, and 120 min. (b) Desorption of CR from FF-LM and FF-SM with DIW, ethanol, 0.1 M sodium hydroxide, 0.1 M acetic acid, 0.1 M hydrochloric acid, and concentrated hydrochloric acid.

The removal efficiency of CR from the simulated dye effluent was found to be >95% for FF-LM, irrespective of the presence or absence of NaCl, as shown in Figure 7a. However, a strong increase in the removal efficiency with increasing NaCl was obtained for FF-SM, reaching about 99.9% at 1 M NaCl. Such an increase in the removal efficiency can be explained by the neutralization of the negatively charged dye molecules and cell wall surface by NaCl.^45^ Overall, these results suggest that the dried mycelium of *F. fomentarius* can be considered a good adsorbent for anionic dyes such as CR from industrial wastewater, even at high salt concentrations.

### Desorption

The results of the desorption experiments of CR from FF-LM and FF-SM using different desorbing agents, that is, deionized water (DIW), ethanol, 0.1 M sodium hydroxide, 0.1 M acetic acid, 0.1 M hydrochloric acid, and concentrated hydrochloric acid, are shown in Figure 7b. No desorption of CR was observed with a diluted concentration (0.1 M) of acetic acid or hydrochloric acid. Although concentrated hydrochloric acid resulted in the highest recovery efficiencies for CR, it also caused the dissolution of *F. fomentarius* mycelia. The results suggest that both organic and mineral acids are not suitable desorbing agents for such fungal adsorbents. However, 0.1 M sodium hydroxide and ethanol could desorb CR from the cell wall surfaces, albeit with low efficiencies of <25%. These observations indicate strong chemical interactions between the functional groups of the fungal cell wall surface and the adsorbed CR. However, biodegradation by composting might be a promising alternative.

### X-Ray Photoelectron Spectroscopy Study of MB and CR Adsorption

**Figure 8:**
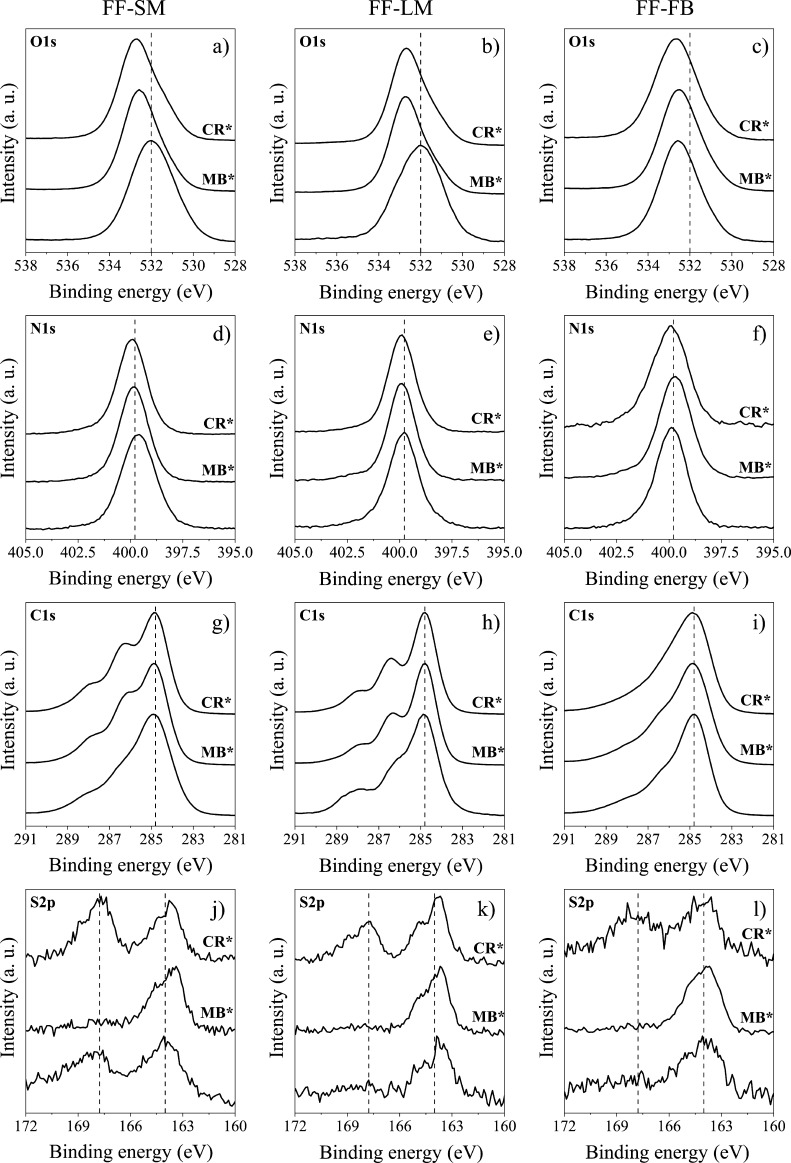
Normalized XPS for FF-SM, FF-LM, and FF-FB before and after (MB* and CR*) adsorption for O 1s (a–c), N 1s (d–f), C 1s (g–i), and S 2p (j–l).

To understand the adsorption mechanism of MB and CR onto FF-LM, FF-FB, and FF-SM, all three samples were characterized by X-ray photoelectron spectroscopy (XPS) analysis before and after the adsorption process. Table S1 summarizes the chemical composition of the specimens, revealing an elevated nitrogen content for FF-LM and thus pointing toward a higher chitin level in the cell wall, as already deduced from the ATR–FTIR results. Figure 8 displays the XPS O 1s, N 1s, C 1s, and S 2p spectra of the three samples before and after the adsorption of MB and CR. It can be seen that O 1s and N 1s XPS peaks shifted to higher binding energies after MB and CR adsorption. As the highest shifts were observed for the O 1s peaks, it can be assumed that strong chemical interactions exist between the oxygenated functional groups on the fungal cell surface and the dyes. This finding was also confirmed by the increase in the peak intensity of −C–O– (286.3 eV), −C=O (288.2 eV), and −O–C=O (289.1 eV) in the C 1s spectra in comparison with that of–C–C– (284.8 eV) after the adsorption of the dyes.^53^ Moreover, after CR adsorption, the S 2p spectra show an increase in intensity for the peak at 168 eV, which is attributed to −C–S=O present in the sulfonate group of CR. Overall, these findings suggest that the adsorption of MB and CR on *F. fomentarius* can be attributed to both electrostatic attraction and chemical interaction, which is in agreement with the results of the isotherm and desorption analyses.

## Conclusions

The performance of the lab-cultivated mycelium from *F. fomentarius*, grown on a solid lignocellulose medium (FF-SM) and a liquid glucose medium (FF-LM), for the adsorption of cationic MB and anionic CR from aqueous solutions was compared with that of the naturally grown fruiting bodies of *F. fomentarius* (FF-FB). While FF-FB showed the lowest dye uptake for both dyes, FF-LM demonstrated the highest CR uptake and a good MB uptake and FF-SM the highest MB uptake with a moderate CR uptake. The adsorption process of CR, which was studied in more detail, could be explained by the Redlich–Peterson model with β constants very close to 1, that is, approaching the Langmuir model and thus indicating monolayer adsorption. FF-LM and FF-SM reached the maximum adsorption capacities of 48.8 and 13.4 mg g^–1^, respectively, for CR at pH 7.6. Adsorption kinetics were found to follow the pseudo-second-order model, implying that the dye adsorption rate was controlled by a chemisorption step. Due to strong interactions, desorption efficiencies of the dyes were below 25%, which requires further studies to improve the desorption efficiency or tests for the suitability for composting. Remarkably, the adsorption performances were preserved under simulated dye effluent conditions for lab-cultivated *F. fomentarius*. Several characterizations, such as XPS, FTIR spectroscopy, and zeta potential analysis, suggest that the amide and amino groups of chitin from the cell walls of *F. fomentarius* play a dominant role in the adsorption process of CR. In contrast, the MB uptake was found to be independent of the chitin content on the surface, indicating that a different adsorption mechanism is dominant. However, further, more detailed studies are required due to the complex chemical structure of the specimens studied in the present work. The overall superiority of the lab-cultivated mycelium over the naturally grown *F. fomentarius* offers several advantages, such as simple and low-cost mass production. Currently, the lab-scale production of a square meter mycelium mat requires 5 weeks of time and ca. 30 € in chemicals for FF-LM and 7 weeks of time and ca. 1 € in chemicals for FF-SM, making FF-SM more economically competitive and interesting for cationic dye adsorption, in particular. Overall, *F. fomentarius* can be considered a promising candidate as a future biosorbent material in industrial wastewater treatment with the opportunity for further improvement.

## Experimental Section

### Chemicals

MB (98%), CR (82%), calcium sulfate dihydrate (98%), and streptomycin sulfate salt were obtained from Sigma-Aldrich (Germany, USA). Hydrochloric acid (37%), acetic acid (100%), sodium chloride (≥95%), malt extract agar, sodium nitrate (>99%), and glucose monohydrate (microbiology grade) were purchased from Carl Roth GmbH + Co. KG (Germany). Sodium hydroxide (p.a.) was obtained from Merck. Ethanol (≥96%), formic acid (≥99%, HiPerSolv CHROMANORM), and acetonitrile (≥99.9%, HiPerSolv CHROMANORM) were provided by VWR (Germany). Ampicillin sodium salt was acquired from AppliChem GmbH (Germany). Hemp shives were purchased from Hemparade (The Netherlands). Brown millet was provided by Mühle Schlingemann (Germany). Yeast extract and Gibco casamino acids were obtained from Ohly GmbH (Germany) and Life Technologies (USA), respectively. DIW was used for stock solution and fungi preparation.

### *F. fomentarius* Cultivation

A fruiting body of *F. fomentarius* was collected from the Brandenburg forest, cut into slices using a band saw (REKORD Typ SSF/420, Maschinenfabrik August Mössner KG, Germany), and stored in a freezer at −18 °C. Since the fruiting body consists of different layers, only the fibrous trama layer^54^ was used. The slices were punched into discs with a diameter of 8 mm and dried at 80 °C for at least 72 h. This fruiting body of *F. fomentarius* collected from the nature is referred to as FF-FB.

An axenic culture derived from a fruiting body was obtained on malt extract agar. After 10 days of growth, colonies were scraped off with a sterile scalpel, DNA extracted as previously described,^55^ and verified by the sequencing of the internal transcribed spacer region located in the rRNA gene transcription region of *F. fomentarius*. The strain was named PaPf11. For emersed cultivation on a a liquid medium, fungal complete medium (CM) with 1% glucose as the carbon source and 70 mM sodium nitrate as the nitrogen source were used.^56^ To the medium, 50 mg L^–1^ ampicillin and 50 mg L^–1^ streptomycin were added to reduce the risk of contamination. The fungal inoculum was obtained by using a sterile electric hand blender (Mixino 260, Siemens, Germany) for about 30 s to shred PaPf11 colonies scraped off from malt extract agar and subsequently transferred into 1 L of CM into smaller entities with diameters of 2 mm or less. This mixture was incubated in a closed, disinfected polypropylene box for 18–20 days in the dark at 25 °C. Mycelium mats of about 5 mm thickness were harvested from the surface of CM and dried on parchment paper at 50 °C for 2 days. The mats were punched into discs with a diameter of 8 mm and dried at 80 °C for at least 72 h. This *F. fomentarius* grown on a liquid glucose medium is referred to as FF-LM.

The protocol for emersed cultivation on a solid medium was recently described in detail and can briefly be summarized as follows.^38^ Colonies of *F. fomentarius* were obtained through cultivation on solid CM and used to inoculate sterilized millet grains supplemented with 1 wt % calcium sulfate dihydrate. After incubation for 14 days at 25 °C in the dark, the grains were completely overgrown by the fungal mycelium and used as the mushroom spawn to inoculate hemp shives composed of lignocellulose as a solid medium. Therefore, hemp shives were hydrated with 150 wt % DIW in polypropylene cultivation bags (SacO2, Belgium) and autoclaved. 5 wt % overgrown millet spawn was added to the wet hemp shives and mixed by kneading. The bags were then heat-sealed and incubated at 25 °C in the dark. After 7 days of incubation, the bags were mixed, and incubation was continued for another 7 days. The overgrown solid substrate was then crushed using a disinfected shredder (AXT Rapid 2000, Bosch, Germany) and transferred into a disinfected polypropylene box. After another 19 days of incubation, the culture was removed from the box and dried at 50 °C for 3 days. Finally, the pure surface mycelium was obtained by careful stripping from the surface. The mats were punched into discs with a diameter of 8 mm and dried additionally at 80 °C for at least 72 h. This *F. fomentarius* grown on a solid lignocellulose medium is referred to as FF-SM.

### Dye Adsorption Experiments

#### Batch Equilibrium Studies

Aqueous stock solutions of cationic MB and anionic CR with a concentration of 1000 mg L^–1^ were prepared by dissolving 1 g of the dye in 1 L of DIW. Solutions with lower dye concentrations were diluted from this stock solution. Adsorption experiments were performed in 50 mL centrifuge tubes with 10 mL of the dye solution at 25 °C and at a steady rotation of 80 rpm in a mixer (Rotator, neoLab, Germany). In the first batch adsorption experiments, FF-SM, FF-LM, and FF-FB were tested with a dosage of 5 g L^–1^ with an initial dye concentration of 100 mg L^–1^ at pH values of 5.7 (MB) and 7.6 (CR) for 120 min in technical triplicate. Further experiments were conducted to study the influence of dosage, time, initial dye concentration, and salts on the adsorption process on FF-SM and FF-LM with CR. The effect of the adsorbent dosage was studied in the range of 0.5–30 g L^–1^. The pH was adjusted between 2.1 and 12.6 by HCl and NaOH. Following the adsorption experiments, the supernatant was decanted and separated from the remaining mycelial by centrifugation at 3600 rpm in a Jouan C414 for 20 min. The dye concentration in the supernatant was determined by UV–vis spectroscopy from the absorption bands at 664 and 498 nm, specific for MB and CR, respectively. The removal efficiency *R*_e_ (%) and the equilibrium adsorption capacity *q*_e_ (mg g^–1^) were calculated as given in eqs 1 and 2, respectively,
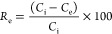
1
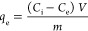
2whereby *C*_i_ (mg L^–1^) and *C*_e_ (mg L^–1^) are the initial and equilibrium mass concentrations of the dye in the solution, *m* (g) is the mass of the adsorbent, and *V* (L) is the volume of the dye solution.

#### Adsorption Isotherms

CR concentrations between 5 and 400 mg L^–1^ were used to obtain the adsorption isotherms. The dosage and contact time were 5 g L^–1^ and 120 min, respectively, for both FF-SM and FF-LM. Mixing and separation were performed as described above. The experimental adsorption data were fitted using the nonlinear form of the Langmuir, Freundlich, and Redlich–Peterson isotherm models expressed by eqs 3–5, respectively,
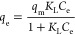
3
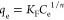
4
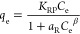
5whereby *q*_e_ (mg g^–1^) is the equilibrium adsorption capacity, *C*_e_ (mg L^–1^) is the equilibrium dye concentration, *q*_m_ (mg g^–1^) is the maximum adsorption capacity, *K*_L_ (L mg^–1^) is the Langmuir isotherm constant, *K*_F_ [mg g^–1^ (L mg^–1^)^1/*n*^] and *n* (mg g^–1^) are the Freundlich coefficients, and *K*_RP_ (L g^–1^), *a*_R_ (L mg^–1^), and β are the Redlich–Peterson constants.

#### Adsorption Kinetics

To study the adsorption kinetics, contact times between 2 and 300 min were tested. The dosage and initial CR concentration were 5 g L^–1^ and 100 mg L^–1^, respectively, for both FF-SM and FF-LM. Mixing and separation were performed as described above. To analyze the adsorption kinetics, the pseudo-first-order model (eq 6), pseudo-second-order model (eq 7), and Elovich model (eq 8) were applied
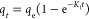
6
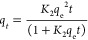
7
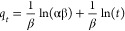
8whereby *t* (min) is the time, *K*_1_ (min^–1^) and *K*_2_ (g mg^–1^ min^–1^) are the pseudo-first-order and pseudo-second-order constants, respectively, and α and β are the initial adsorption rate and the Elovich desorption constant, respectively.

#### Adsorption Behavior under Simulated Textile Effluent Conditions

To simulate the textile effluent conditions, adsorption experiments were conducted at elevated temperature, that is, at 80 °C, in the presence of salts (0, 0.01, 0.1, and 1 M NaCl). The dosage, pH, and CR concentration were 5 g L^–1^, 7.6, and 100 mg L^–1^, respectively, for both FF-LM and FF-SM. Experiments were conducted in covered 50 mL beakers on a hot plate under stirring at 80 rpm for 120 min.

### Dye Recovery from the Adsorbents

Batch mode adsorption was performed as described above with a dosage of 5 g L^–1^ for both FF-LM and FF-SM. The adsorbents were then dried at 60 °C overnight before 10 mL of the desorption agents, that is, DIW, ethanol, 0.1 M NaOH, 0.1 M acetic acid, 0.1 M HCl, or concentrated HCl, was added. Mixing was performed in 50 mL centrifuge tubes for 120 min at 25 °C at a steady rotation of 80 rpm in a mixer (Rotator, neoLab, Germany).

### Characterization

All *F. fomentarius* samples, that is, FF-SM, FF-LM, and FF-FB, were characterized with the methods described below unless stated differently.

The dye concentration was determined by UV–vis spectroscopy from the absorption bands at 664 and 498 nm, specific for MB and CR, respectively, using a Lambda 900 (Perkin Elmer, USA).

FTIR spectroscopy in the ATR mode was carried out in a Vertex 70 (Bruker, Germany) in the range of 4000–400 cm^–1^ for the identification of distinct functional groups.

The microstructure of the mycelial structures was studied via SEM using a LEO 1530 (Carl Zeiss, Germany) at 3 kV with a secondary electron detector and an aperture size of 30 μm after sputtering with a thin gold layer.

Their zeta potential was determined using an electroacoustic spectrometer DT-310 (Dispersion Technology Inc., USA). Therefore, 0.15 wt % suspensions in 0.01 M KCl were prepared by blending mycelia with a hand blender (Kult S, WMF, Germany). Before the measurement, the samples were treated in an RK 52 H ultrasonic bath (Bandelin, Germany) for 2 min. The sample mass was 50 g per measurement. For both FF-SM and FF-LM, two measurements were conducted toward acidic conditions down to pH 2 and toward basic conditions up to pH 12. Titration was performed using 0.1 M HCl and 0.1 M NaOH solutions. For FF-FB, two measurement points at pH values of 5.7 and 7.6 were recorded, representing the pH of the initial dye solutions of MB and CR, respectively.

XPS spectra were collected on a K-Alpha (Thermo Fischer Scientific, USA) equipped with a monochromatic Al Kα source to obtain the surface chemical composition of the *F. fomentarius* samples. Therefore, the samples were prepared on carbon pads. Scans were recorded in the constant analyzer energy mode with a pass energy of 50 eV, a step size of 0.1 eV, and a spot size of 400 μm. All XPS spectra were calibrated using the C 1s core line with a binding energy of 284.8 eV.

HPLC–electrospray ionization–MS was conducted on an Agilent 1200 series HPLC system using a reversed-phase HPLC column (Grom-Sil-120-ODS-4-HE, length 50 mm, ID 2 mm, 3 μm, Dr. Maisch, Germany) coupled to an LTQ Orbitrap XL (Thermo Fischer Scientific, USA) in the positive ionization mode. A gradient of mobile phase A (water + 0.1% formic acid) and B (acetonitrile + 0.1% formic acid) was applied to change from 5 to 100% B in 10 min at a flow rate of 0.3 mL min^–1^. During the LC separation, the column effluent was additionally scanned for the UV absorbance with the integrated diode array detector G1313B of the LC system. The resulting *m*/*z* scan and UV–vis absorbance data were evaluated using an Xcalibur (Thermo Fisher Scientific, USA).

## Supporting Information Available

Photographs of supernatants after dye adsorption; photographs and UV–vis spectra of supernatants after the contact of *F. fomentarius* with aqueous solutions of varying pH; HPLC-MS results; and XPS chemical composition (PDF)
