# Supplementary material for: Effect of Fomes fomentarius Cultivation Conditions on Its Adsorption Performance for Anionic and Cationic Dyes
Source: ACS Omega. 2022 Jan 24;7(5):4158–69. doi: 10.1021/acsomega.1c05748 (PMC8829953; doi:10.1021/acsomega.1c05748)
Supplement: Supplementary file 1 — ao1c05748_si_001.pdf [file ao1c05748_si_001.pdf]

Supporting information

Effect of *Fomes fomentarius* cultivation conditions on its adsorption performance for anionic and cationic dyes

Laura M. Henning[a]*, Ulla Simon[a], Amanmyrat Abdullayev[a], Bertram Schmidt[b], Carsten Pohl[b], Tamara Nunez Guitar[b], Cekdar Vakifahmetoglu[c], Vera Meyer[b], Maged F. Bekheet[a]*, Aleksander Gurlo[a]

[a] Technische Universität Berlin, Faculty III Process Sciences, Institute of Material Science and Technology, Chair of Advanced Ceramic Materials, Straße des 17. Juni 135, 10623 Berlin, Germany

[b] Technische Universität Berlin, Faculty III Process Sciences, Institute of Biotechnology, Chair of Applied and Molecular Microbiology, Straße des 17. Juni 135, 10623 Berlin, Germany

[c] Izmir Institute of Technology, Department of Materials Science and Engineering, 35430, Urla, Izmir, Turkey

* corresponding authors: Laura M. Henning, laura.m.henning@ceramics.tu-berlin.de, +49 (0) 30 314 70483, Maged F. Bekheet, maged.bekheet@ceramics.tu-berlin.de, +49 (0) 30 314 22591

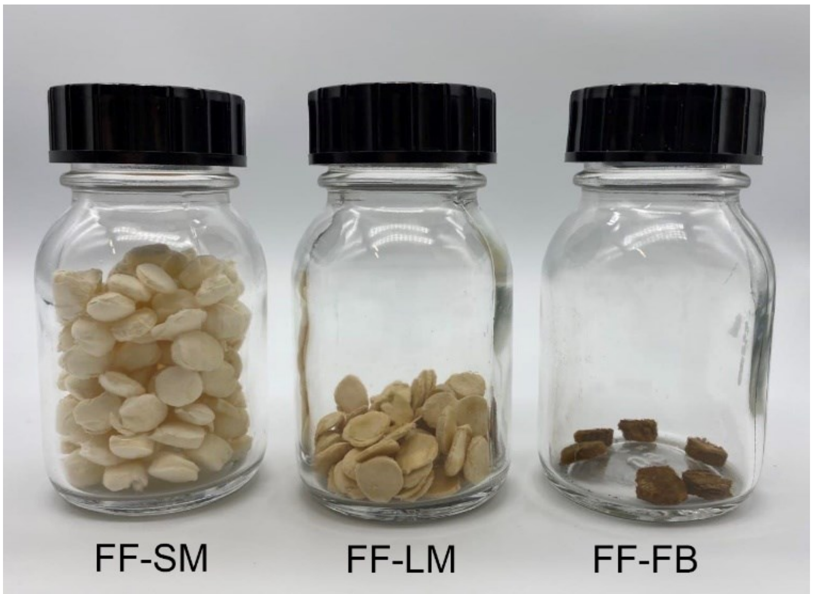

Figure S1. Discs with a diameter of ca. 8 mm, each ca. 0.3 g, from left to right, from *F. fomentarius* cultivated on solid lignocellulose medium (FF-SM), cultivated on liquid glucose medium (FF-LM), and obtained as fruiting body from a tree (FF-FB).

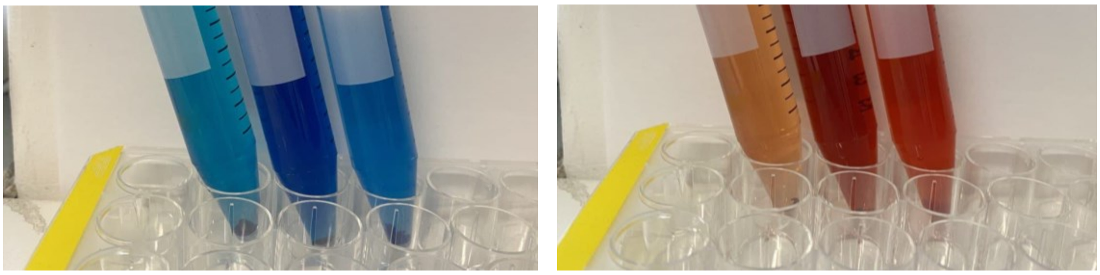

Figure S2. Photographs of supernatants after the adsorption of MB (left) and CR (right) on FF-SM, on FF-LM, and FF-FB. Adsorption conditions were 5 g L^{-1}, 100 mg L^{-1} MB and CR, pH 5.7 (MB), 7.6 (CR), 120 min.

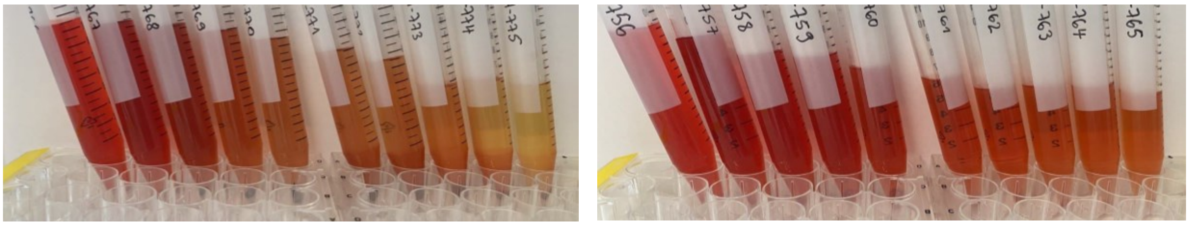

Figure S3. Photographs of supernatants showing the effect of adsorbent dosage on the removal efficiency and thus, decoloring of CR on FF-LM (left) and FF-SM (right). Adsorption conditions were pH 7.6, 100 mg L^{-1}, 120 min.

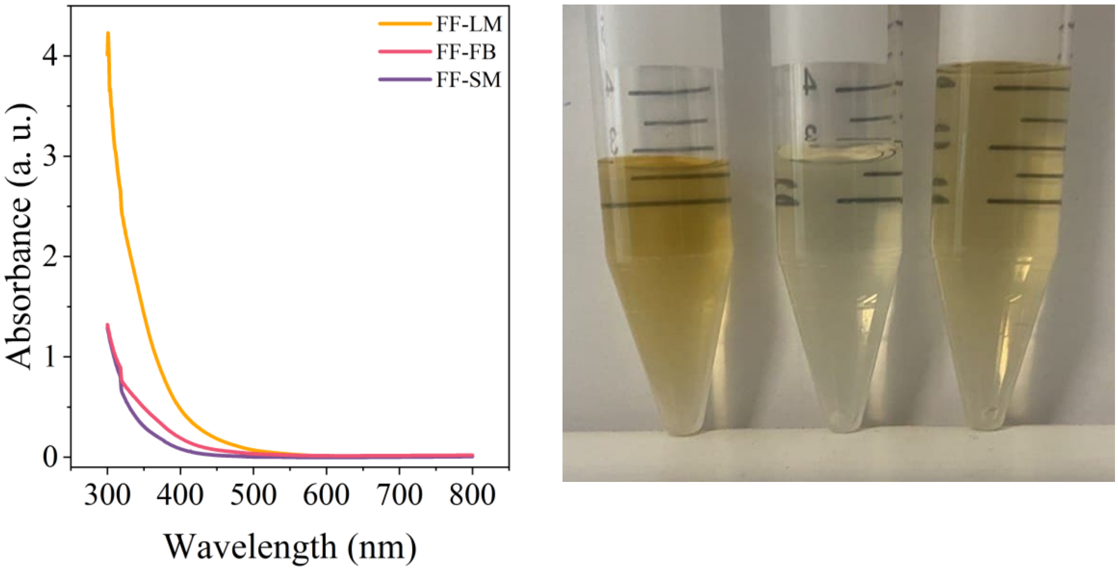

Figure S4. Coloring of DIW by different types of fungi: Left: UV-Vis spectrum of the supernatants of FF-LM, FF-FB, and FF-SM after 120 min mixing with DIW. Dosage was 30 g L^{-1}. Right: Photographs of the corresponding supernatants, from left to right: FF-LM, FF-SM, FF-FB.

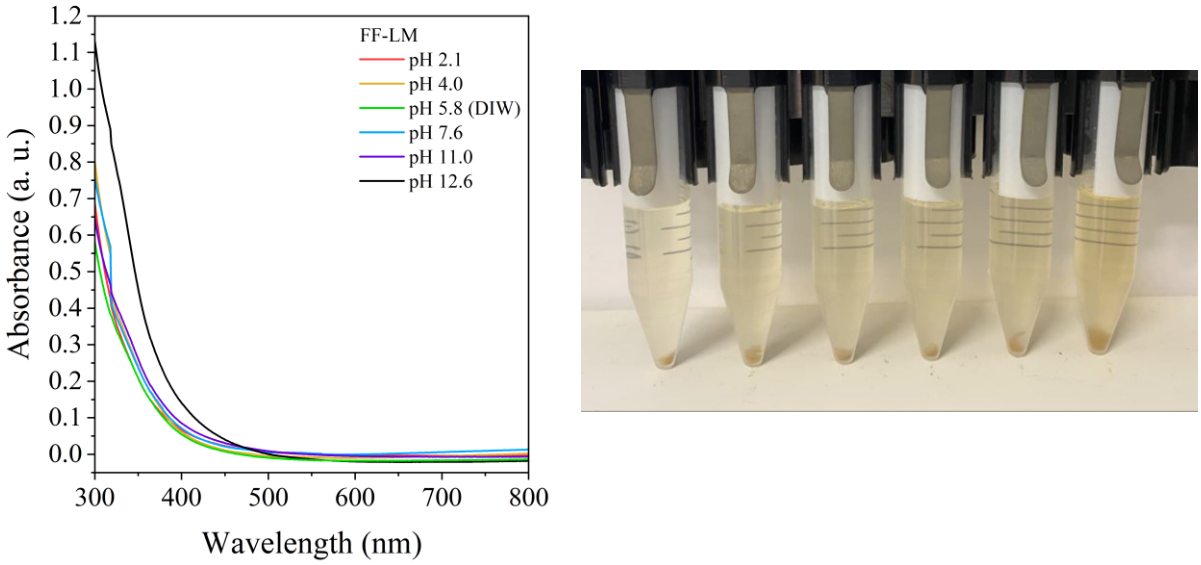

Figure S5. Coloring of DIW by FF-LM depending on the pH. Left: UV-Vis spectrum of the supernatants after 120 min mixing with DIW/NaOH/ HCl for pH 2.1, 4.0, 5.8, 7.6, 11.0, 12.6. Dosage was 5 g L^{-1}, volume of 5 mL. Right: Photographs of the corresponding supernatants, from left to right with increasing pH (2.1, 4.0, 5.8, 7.6, 11.0, 12.6).

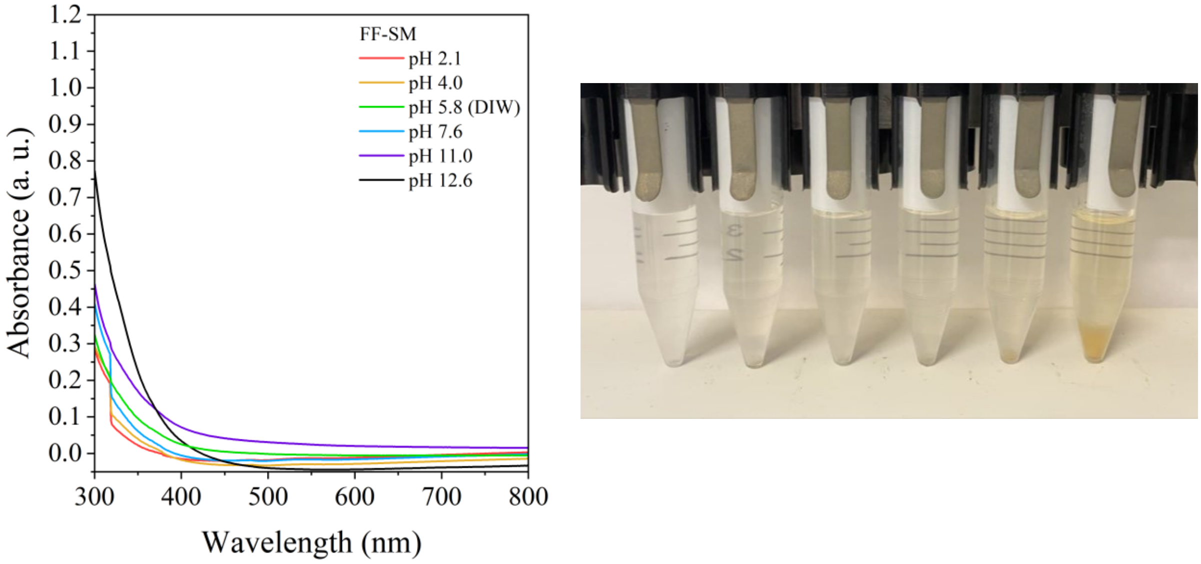

Figure S6. Coloring of DIW by FF-SM depending on the pH. Left: UV-vis spectrum of the supernatants of FF-LM after 120 min mixing with DIW/NaOH/ HCl for pH 2.1, 4.0, 5.8, 7.6, 11.0, 12.6. Dosage was 5 g L^{-1}, volume of 5 mL. Right: Photographs of the according supernatants, from left to right with increasing pH (2.1, 4.0, 5.8, 7.6, 11.0, 12.6).

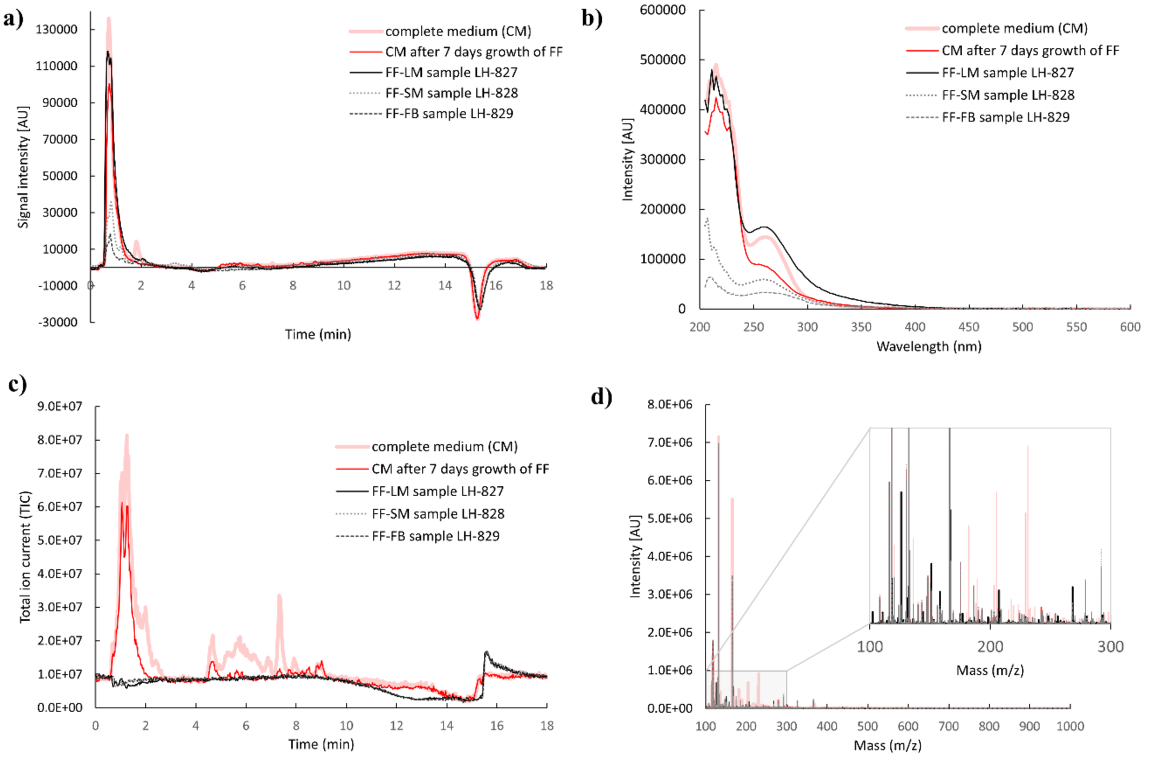

Figure S7. Results of HPLC-MS analysis of complete medium (CM), CM after 7 days of growth with *F. fomentarius* in liquid culture and clear supernatants from FF-LM, FF-SM, and FF-FB (30 g L^{-1} in DIW). a) Total PDA signal intensity across sample separation run indicates early eluting hydrophilic compounds are the main contributors to the signal. b) Accumulated wavelength spectra from 200 to 600 nm in a 0.5 to 2.0 min run time window show absorption peaks at 210 and 260 nm. c) Total ion current of samples recorded from 100 to 1000 m/z. d) Accumulated m/z in a 0.5 to 2.0 min run time window. The insert shows the range of 100 to 300 m/z for clarity.

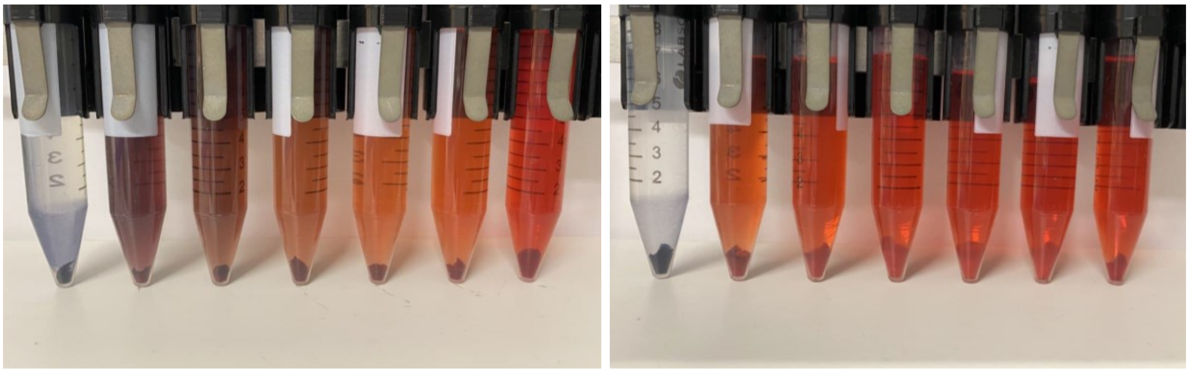

Figure S8. Photographs of the effect of pH (acidic to basic, from left to right) on the adsorption of CR on FF-LM (left) and FF-SM (right). Adsorption conditions were pH = 7.6, C_i = 100 mg L^{-1}, t = 120 min.

Table S1: Chemical composition of the surface of FF-SM, FF-LM, and FF-FB before and after dye adsorption as revealed by XPS.

**Sample**	**O 1s (at%)**	**N1s (at%)**	**C 1s (at%)**	**S 2p (at%)**	**Others (at%)**
FF-SM	23.08	4.9	71.12	0.32	0.59
FF-SM-MB*	22.95	5.13	71.65	0.27	< 0.1
FF-SM-CR*	23.18	5.33	70.78	0.27	0.45
FF-LM	24.32	8.89	64.84	0.26	1.69
FF-LM-MB*	21.27	3.85	74.23	0.25	0.39
FF-LM-CR*	22.34	4.01	72.79	0.26	0.6
FF-FB	20.23	3.37	76.13	0.15	0.12
FF-FB-MB*	21.13	3.32	75.22	0.34	< 0.1
FF-FB-CR*	18.74	2.54	78.46	0.13	0.13
